# Rethinking dynamics: static amino acid PET parameters vs. dynamic amino acid PET parameters for the detection of tumor progression in patients with post-treatment glioma

**DOI:** 10.3389/fnume.2026.1762984

**Published:** 2026-02-18

**Authors:** Dylan Henssen, Michael Rullmann, Andreas Schildan, Stephan Striepe, Matti Schürer, Cordula Scherlach, Katja Jähne, Ruth Stassart, Osama Sabri, Clemens Seidel, Swen Hesse

**Affiliations:** 1 Department of Nuclear Medicine, University Hospital Leipzig, Leipzig, Germany; 2 Department of Medical Imaging, Radboud University Medical Center, Nijmegen, Netherlands; 3 Department of Radiation Oncology, University Hospital Leipzig, Leipzig, Germany; 4 Institute for Neuroradiology, University Hospital Leipzig, Leipzig, Germany; 5 Department of Neurosurgery, University Hospital Leipzig, Leipzig, Germany; 6 Institute of Neuropathology, University of Leipzig, Leipzig, Germany

**Keywords:** amino acid PET, molecular imaging, neuro-oncologic emergency, PET-MRI, pseudoprogression, radionecrosis

## Abstract

**Background:**

It remains unclear whether dynamic amino-acid (AA) positron-emission tomography (PET) has additional diagnostic value over static AA-PET to distinguish tumor progression (TP) from treatment-related abnormalities (TRA) in patients with post-treatment glioma.

**Methods:**

This was a retrospective study of patients with glioma with suspected TP who underwent dynamic AA-PET imaging. The final diagnoses were based on histopathology and/or clinical-radiological follow-up. The static PET parameters included the mean and maximum tumor-to-brain ratio (TBR_max_ and TBR_mean_, respectively) and the dynamic PET parameters included time to peak (TTP) and area under the time activity curve (AUTAC). Diagnostic accuracy was assessed using the area under the receiver operating characteristic curve (AUROC).

**Results:**

In total, 33 patients with adult diffuse glioma (17 females: mean age: 55.7 ± 12.2 years) were included [13 [S-methyl-^11^C]methionine ([^11^C]MET) and 20 O-(2-[^18^F]fluoroethyl)-L-tyrosine (^1^⁸F]FET) PET examinations]. The static parameters (TBR_mean_ and TBR_max_) were significantly different between the TP and TRA groups when using [^11^C]MET (*p* = 0.019 and *p* = 0.013, respectively), resulting in very-good-to-excellent diagnostic accuracy (AUROC values of 0.85 and 0.93, respectively). The TBR_mean_ values derived from [^18^F]FET PET data were not significantly different between the TP and TRA groups (*p* = 0.066). However, the [^18^F]FET PET data-derived TBR_max_ values were significantly higher in the individuals with TP (*p* = 0.005), indicating very good diagnostic accuracy (AUROC = 0.84). The dynamic PET parameters (time to peak and area under the time activity curve) were not significantly different between the TP and TRA groups.

**Conclusion:**

This study suggests that dynamic and static AA-PET parameters have similar diagnostic capacities to distinguish TP from TRA. While static AA-PET parameters may suffice for clinical decision-making, this study did not formally assess the incremental value of using dynamic metrics in addition to static measures.

## Introduction

Diffuse infiltrating gliomas can have an astrocytic or oligodendroglial origin—World Health Organization (WHO) grades 2–4, depending on subtype—and have a high morbidity and mortality even with optimal treatment consisting of surgical resection and postoperative chemoradiotherapy (1). This is due to the (microscopic) infiltrative growth pattern of glioma and frequent treatment resistance, which in turn leads to frequently observed post-treatment tumor progression (TP), i.e., the renewed occurrence or progression of enhancing areas within the remaining tumor or surgical bed on follow-up conventional magnetic resonance imaging (MRI). However, treatment-related abnormalities (TRA), including pseudoprogression and radiation necrosis, have almost identical characteristics on conventional MRI (2), resulting in a diagnostic challenge (3, 4). Considering the fact that both entities require vastly different therapeutic approaches and are associated with significantly different outcomes, more sophisticated imaging techniques have been proposed to distinguish TP from TRA in recent years (5–7). These techniques include advanced MRI techniques (e.g., diffusion-weighted MRI, perfusion-weighted MRI, and MR spectroscopy) and positron emission tomography (PET) imaging with radiolabeled amino acids (AA-PET). Despite a histopathological evaluation of tissue obtained through a biopsy or resection being the gold-standard diagnostic approach, there is a medical need for highly accurate non-invasive techniques. Molecular imaging reveals the physiological properties of the tissue non-invasively and is therefore regarded as the most optimal non-invasive technique to differentiate TP from TRA (8). In general, it is thought that static PET imaging provides sufficient information when used in the postoperative setting in order to discern TP from TRA. However, static PET imaging does not capture all the biological properties of either TP or TRA lesions and dynamic imaging protocols have been developed, especially for O-(2-[^18^F]fluoroethyl)-L-tyrosine ([^18^F]FET)-PET imaging (9). The clinical value of such dynamic imaging protocols, in comparison with static imaging protocols, however, remains unclear. This study, therefore, investigated the diagnostic accuracy of static vs. dynamic AA-PET in distinguishing between TP and TRA in patients with post-treatment glioma.

## Materials and methods

### Ethical approval

The local medical ethical committee at our hospital approved this study (ethical review board assigned file number: 014/21-ek). All the patients provided written informed consent to undergo the PET imaging protocol.

### Included patients

Patients with post-treatment adult-type diffuse glioma (≥18 years old) were eligible for inclusion in this study. Patients were included when follow-up MRI revealed a new contrast-enhancing lesion of undetermined etiology (i.e., reflecting either TP or TRA). All the patients underwent a dynamic AA-PET imaging protocol using either [S-methyl-^11^C]methionine ([^11^C]MET) or [^18^F]FET. To ascertain the outcome, two methods were used. The first was the gold-standard diagnostic approach, i.e., a histopathological evaluation of resected/biopsied tissue obtained from the new contrast-enhancing lesion (*n* = 8). When a histopathological examination was not available, the second method was clinical and radiological follow-up over a period of at least 6 months, following the criteria published by the Response Assessment in Neuro-Oncology working group (10). Mixed lesions, i.e., lesions for which histopathological assessment or clinico-radiological follow-up provided no preferable, definite diagnosis, were excluded from this study.

### Radiosynthesis of [^11^C]MET and [^18^F]FET

**Figure 1:**
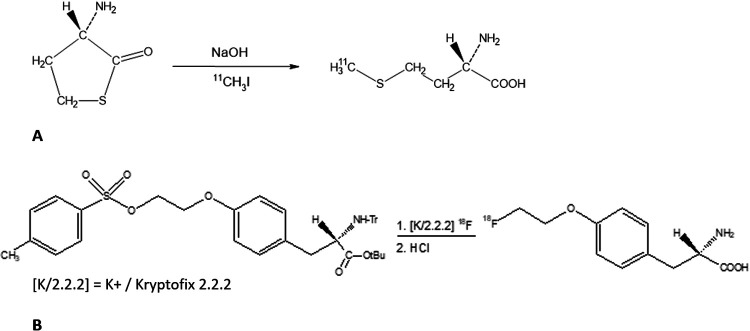
Reaction mechanism for the radiosynthesis of (**A**) [^11^C]MET and (**B**) [^18^F]FET.

The synthesis of [^11^C]MET proceeds according to the reaction scheme presented in Figure 1A. [^11^C]methyl iodide is produced using a methyl iodide Microlab synthesis module (GE Healthcare, USA). The [^11^C]methyl iodide is then passed through a stainless steel reaction loop of a modified TRACERlab FX synthesis module (GE Healthcare) at room temperature, into which a solution of L-homocysteine thiolactone hydrochloride in sodium hydroxide and ethanol is injected. After completion of the transfer of [^11^C]methyl iodide, the reaction mixture, with water and ethanol added, is passed through a combination of solid phase extraction cartridges. The [^11^C]MET is fixed on an anion exchange cartridge and eluted with a di-sodium hydrogen phosphate solution. The eluate is then formulated into the final product using sodium phosphate buffer and hydrochloric acid.

Irradiated enriched [^18^O]water with [^18^F]fluoride is transferred to an All-in-One Synthesis Module (Trasis, USA). The [^18^F]fluoride is then separated from the ^18^O-containing water by adsorption on an anion exchanger. The synthesis of [^18^F]FET then proceeds according to the reaction scheme (Figure 1B). The [^18^F]fluoride that is fixed on the anion exchanger is eluted into the reaction vessel using an aqueous solution of potassium carbonate, Kryptofix, and acetonitrile. After azeotropic drying of the mixture, precursor (2S)-O-(2ʹ-tosyloxyethyl)-*N*-trityl-tyrosine-*tert*-butyl ester (TET) in dry acetonitrile is added. The reaction vessel is then heated, during which the nucleophilic substitution takes place. After evaporation of the solvent in a vacuum, the intermediate product is hydrolyzed by adding hydrochloric acid and finally fixed on solid phase extraction cartridges. The solid phase extraction cartridges are eluted with an ethanol/water mixture. After buffering with citrate buffer, the product is transferred via a preconditioned Alumina-N light cartridge into a sterile bulk container.

### PET imaging protocol and post-processing of PET data

All dynamic PET-MRI data were acquired using a 3T PET-MRI system (Siemens Biograph mMR, Siemens Healthineers, Erlangen, Germany). After an intravenous bolus injection of one of the radiolabeled amino acids, dynamic PET data were acquired in 3D list mode from 0 to 60 min. The emission recording reconstructions consisted of 38 time frames (time frames 1–12: 15 s each, time frames 13–19: 30 s each, time frames 20–24: 60 s each, time frames 25–29: 120 s each, time frames 30–34: 180 s each, time frames 35–36: 300 s each, time frames 37–38: 600 s each), covering the entire list-mode scan duration up to 60 min after the injection. Images were reconstructed using a 256 × 256 matrix (voxel size 1.00 × 1.00 × 2.03 mm^3^) using a default subset expectation maximization algorithm with eight iterations, 21 subsets, and a 3 mm Gaussian smoothing filter. For attenuation correction, the HiRES method was used. This method combines the individual Dixon attenuation correction approach with a bone attenuation template.

**Figure 2:**
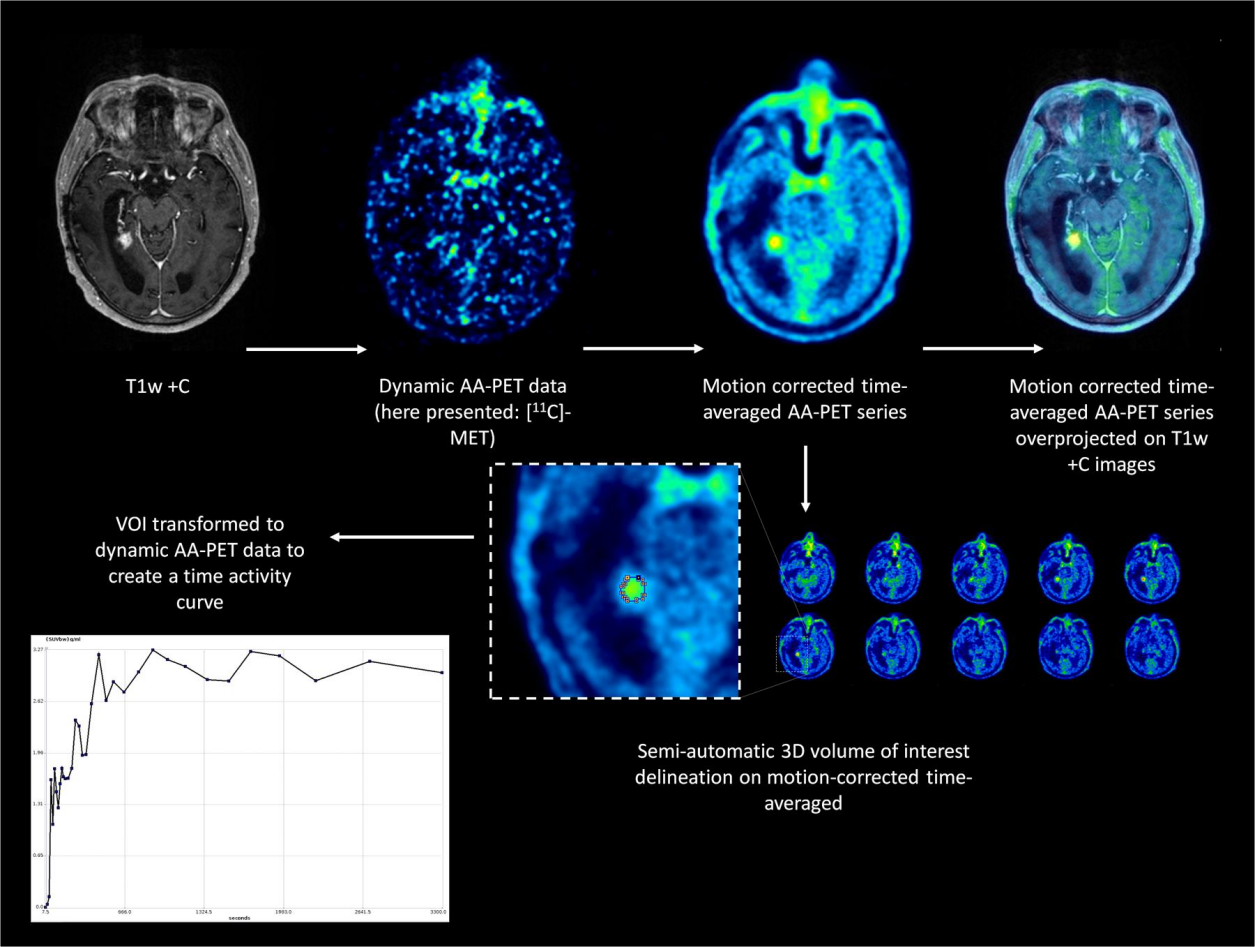
Dynamic AA-PET analysis methodology. Following the intravenous injection of either [^11^C]MET or [^1^⁸F]FET, dynamic amino-acid PET data were acquired continuously for 60 min. Motion correction and co-registration of PET with anatomical T1-weighted MRI were performed using PMOD software. Semi-automatic 3D VOI delineation (hot 3D method) was used to segment the new contrast-enhancing lesion, guided by the fused PET-MRI dataset. A reference VOI was positioned over contralateral normal-appearing brain tissue to calculate the tumor-to-brain ratio (TBR). From the dynamic data, both static (TBR_mean_ and TBR_max_) and dynamic (time to peak, TTP) PET parameters were extracted for subsequent statistical analyses.

The dynamic PET data were motion-corrected and co-registered with individual T1-weighted MRI images using PMOD (PMOD Technologies LLC, Zürich, Switzerland). Dynamic PET data from each patient were reconstructed as time-averaged images. These time-averaged images were used for volume of interest (VOI) delineation using the “Hot 3D” semi-automatic segmentation method. Each new contrast-enhancing lesion was delineated by overlaying PET and T1-weighted MRI data (Figure 2). The semi-automatic segmentation method used a threshold of 45% of the maximum value within the VOI. Each segment was inspected visually by one of the researchers [D.H., a board-certified nuclear medicine physician/radiologist with over 10 years of experience with (experimental) neuro-imaging]. Semilunar-shaped VOIs were positioned over contralateral normal-appearing brain tissue. We ensured the inclusion of both cortex and subcortical white matter, following current evidence-based recommendations (9). Furthermore, the VOIs of normal-appearing brain tissue were used to ensure the time activity curves did not suffer from artifacts. Static PET data were derived from the dynamic PET imaging protocol by summing the imaging data acquired 20–40 min post-injection.

Time activity plots and other dynamic PET data were calculated using PMOD (PMOD Technologies LLC, Zürich, Switzerland). No particular fitting and/or smoothing models were used to create the time activity plots. We calculated the time to peak (TTP; s) and area under the time activity curve (AUTAC; SUV·min) (dynamic AA-PET parameters) and the mean and maximum standardized uptake values (SUVs; g/mL) (static AA-PET parameters) for the VOI representing the new contrast-enhancing lesion. For the reference VOI, we calculated the mean SUV (g/mL) in order to calculate the mean and maximum tumor-to-brain ratios (TBRs), following the current guidelines (9). Assessment of the static and dynamic PET parameters was carried out by one of the investigators (DH) who was blinded to the definite diagnosis derived from histopathological assessment or clinico-radiological follow-up.

### Statistical analysis

All statistical analyses were performed on IBM SPSS Statistics for Windows, version 29 (IBM Corp., Armonk, NY, USA). The study population was grouped according to the radiotracer used (i.e., [^11^C]MET or [^18^F]FET). To assess the differences in static and dynamic PET parameters between the TP and TRA groups per radiotracer, the independent Student's *T*-test was used. The statistical analyses for group comparisons were dependent on the data distribution. If normally distributed, the group comparison was carried out using Student's *t*-test. If the data were not normally distributed, the Mann–Whitney *U*-test was used. Group comparisons of categorical data were carried out using Fisher's exact test. *The post hoc* Bonferroni correction was applied per tracer, correcting for the number of metrics tested within each tracer group. The reported *p*-values are Bonferroni-corrected and the level of significance was *P* < 0.05.

To assess the diagnostic accuracy of statistically significant dynamic or static AA-PET metrics, a receiver operating characteristic (ROC) curve was plotted. By calculating the area under the ROC curve (AUROC), the diagnostic accuracy was determined. The AUROC is a measure of diagnostic accuracy and varies from 0.0 to 1.0. An AUROC between 0.5–0.6 is considered unsatisfactory, while an AUROC of 0.6–0.7 is considered satisfactory, an AUROC of 0.7–0.8 is considered good, an AUROC of 0.8–0.9 is considered very good and an AUROC of 0.9 or higher is considered excellent (11, 12). Differences between ROC curves were assessed using the DeLong test. Furthermore, the ROC was used to determine the optimal cut-off value of each metric to distinguish TRA from TP. The ROC curve analyses were carried out using Python (v3.11).

## Results

In total, 33 patients [17 females, mean age of 55.7 ± 12.2 years (standard deviation)] were eligible for inclusion. Five patients were diagnosed with oligodendroglioma (WHO grade 2: 3; WHO grade 3: 2), seven with astrocytoma (WHO grade 2: 5; WHO grade 3: 2), and 21 with glioblastoma (WHO grade 4: 21) at baseline. Moreover, 13 PET-MRI examinations were carried out using [^11^C]MET and 20 examinations were performed after the administration of [^18^F]FET. The mean administered doses of [^11^C]MET and [^18^F]FET were 719 MBq (± 51 MBq) and 209 MBq (± 26 MBq), respectively. A more detailed overview of the included cohort according to tracer is provided in Table 1.

**Table 1:** Demographics of the patients included in this study.

Parameters	[^18^F]FET group (*n* = 20)	[^11^C]MET group (*n* = 13)	Test	*P*-value
Age (years)	57.1 (± 12.8)	53.4 (± 11.2)	Student's *t*-test	0.587
F:M	8:12	9:4	Fisher's exact test	0.436
TP:TRA	14:6	10:3	Fisher's exact test	0.492
TBR_max_	3.0 (± 0.96)	2.5 (± 1.0)	Student's *t*-test	0.585
TBR_mean_	2.4 (± 0.66)	2.1 (± 0.65)	Student's *t*-test	0.879
TTP (s)	75.0 (22.5–315.0)	141.5 (45–315.1)	Mann–Whitney *U*-test	0.255
AUTAC (SUV·s)	1,568.9 (146.8–4,540.8)	1,671.2 (578.9–6,955.6)	Mann–Whitney *U*-test	0.888

AUTAC, area under the time activity curve; [^11^C]MET, [S-methyl-^11^C]methionine; F, female; [^18^F]FET, O-(2-[^18^F]fluoroethyl)-L-tyrosine; M, male; SUV, standardized uptake value; TBR, tumor-to-brain ratio; TTP, time to peak; TP, tumor progression; TRA, treatment-related abnormalities.

No significant differences in patient characteristics and PET parameters were found when comparing the two radiotracer groups (Table 1). This indicated that the static and dynamic variables derived from AA-PET were comparable for the neuro-oncological disorders covered in this study.

The TBR_mean_ and TBR_max_ values were significantly different between TRA and TP lesions when using [^11^C]MET PET (*p* = 0.019 and *p* = 0.013, respectively). The analysis of the TTP in the dynamic [^11^C]MET PET data revealed no significant differences between the TRA and TP groups (*p* = 0.007). Similarly, the analysis of the AUTAC derived from [^11^C]MET PET data showed no significant differences between patients with TRA and those with TP (*p* = 0.121).

When comparing the TBR_mean_ values derived from the [^18^F]FET-PET data, there was no significant difference between the TRA and TP groups (*p* = 0.066). When comparing the TBR_max_ values derived from the [^18^F]FET-PET data between the TRA and TP groups, a significant difference was observed (*p* = 0.005). Furthermore, a significant difference was found in AUTAC values derived from the dynamic [^18^F]FET-PET data between the TP and TRA groups (*p* = 0.002). No significant differences were found between the TP and TRA groups when analyzing the TTP values derived from the dynamic [^18^F]FET-PET data (*p* = 0.271).

The analysis of the [^11^C]MET PET data-derived parameters TBR_mean_ and TBR_max_ indicated in very-good-to-excellent diagnostic accuracy (AUROC = 0.93; 95%-CI: 0.73–1.00 and AUROC = 0.85; 95%-CI: 0.61–1.00, respectively) for the detection of TP. Moreover, the diagnostic accuracy of TTP was found to be very good (AUROC = 0.87; 95%-CI: 0.58–1.00), whereas the AUTAC demonstrated poor diagnostic accuracy (AUROC = 0.47; 95%-CI: 0.00–0.92). Based on the overlapping confidence intervals of the AUC values, none of the metrics was significantly better at distinguishing TP from TRA.

For TBR_mean_, an optimal cut-off value of 1.83 provided a balanced sensitivity and specificity of 80% and 100%, respectively. For TBR_max_, an optimal cut-off value of 2.3 provided a balanced sensitivity and specificity of 70% and 100%, respectively. Furthermore, for TTP, an optimal cut-off value of 141.5 provided a balanced sensitivity and specificity of 70% and 100%, respectively. Finally, for the AUTAC, an optimal cut-off value of 1,671.2 provided a balanced sensitivity and specificity of 60% and 67%, respectively.

The analysis of the [^18^F]FET PET data-derived parameters TBR_mean_ and TBR_max_ indicated a good-to-very-good diagnostic accuracy (AUROC = 0.73; 95%-CI: 0.39–0.98 and AUROC = 0.84; 95%-CI: 0.62–0.99, respectively) for the detection of TP. The diagnostic accuracy of TTP was found to be unsatisfactory (AUROC = 0.54; 95%-CI: 0.24–0.82), whereas the AUTAC demonstrated very good diagnostic accuracy (AUROC = 0.83; 95%-CI: 0.53–1.00). Based on the overlapping confidence intervals of the AUC values, none of the static or dynamic metrics was significantly better at distinguishing TP from TRA.

For TBR_mean_, an optimal cut-off value of 2.36 provided a balanced sensitivity and specificity of 71% and 83%, respectively. For TBR_max_, an optimal cut-off value of 3.3 provided a balanced sensitivity and specificity of 57% and 100%, respectively. Furthermore, for TTP, an optimal cut-off value of 127.5 provided a balanced sensitivity and specificity of 43% and 83%, respectively. Finally, for the AUTAC, an optimal cut-off value of 1,481.1 provided a balanced sensitivity and specificity of 93% and 83%, respectively.

**Figure 3:**
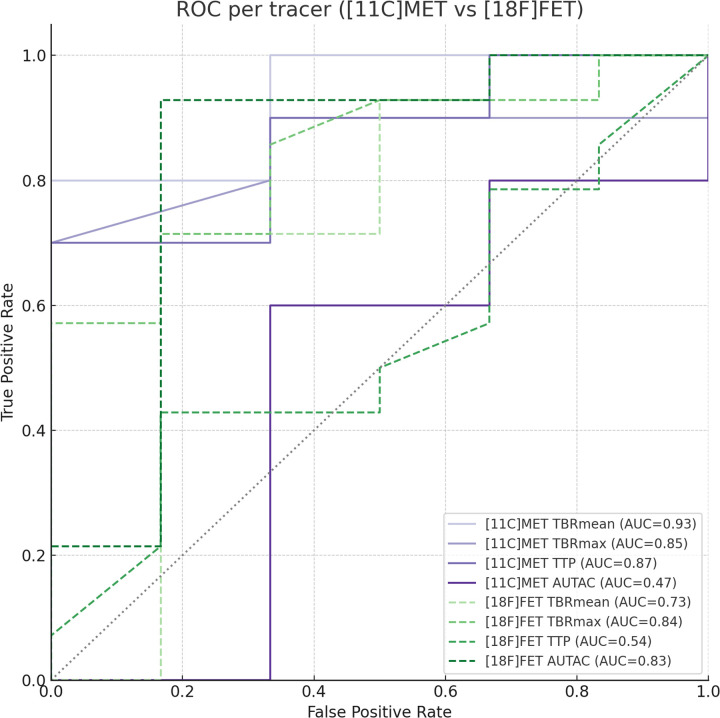
Receiving operator characteristics curves of various parameters derived from AA-PET imaging.

Figure 3 shows the ROC curve for each of the static and dynamic PET parameters that were derived from either [^18^F]FET or [^11^C]MET PET data for the differentiation between TP and TRA.

## Discussion

We found that dynamic AA-PET can provide some additional insights that can help distinguish TP from TRA in patients with post-treatment glioma. However, whether a 60-min dynamic AA-PET examination (especially when using [^11^C]MET) is a necessity remains to be investigated in future studies. In general, a 20-minute static AA-PET examination seems to suffice to distinguish TP from TRA in clinical practice. This is contrary to the results reported by Kebir and colleagues, who found a mean time to peak of 25 min in TP vs. a mean time to peak of 40 min in TRA (*P* < 0.001) (13), as we found no differences in time to peak between patients with TP and TRA. Interestingly, our study shows a much earlier peak in both TP (2.15 min) and TRA (1.95 min) during dynamic AA-PET imaging with [^18^F]FET compared to Kebir et al. (13). This indicates that the vascular phase of the image acquisition plays an important role when using the state-of-the-art imaging equipment detailed in this study. The difference in results can be further explained by differences in the emission recording protocols. Kebir et al. reported the use of 16 time frames, in which the first 5 min are recorded in five time frames of 1 minute each, resulting in a coarser evaluation of early dynamics (13). In our study, however, greater emphasis was placed on the early dynamic phase. This emphasis is also recommended in the most recent international guidelines on this topic (9). This could help explain these findings, though more research is needed to investigate whether this hypothesis holds.

Furthermore, our study shows differences in the diagnostic capacity of TTP and AUTAC for [^18^F]FET and [^11^C]MET PET imaging. Differences in the diagnostic capacity of dynamic PET imaging using [^18^F]FET and [^11^C]MET are already well-known in the pre-treatment imaging setting. In this setting, only the use of [^18^F]FET has an established clinical value in dynamic PET imaging (5). Dynamic [^18^F]FET PET imaging has been reported to increase diagnostic accuracy in tumor grading, as WHO grade 2 gliomas typically show a steadily increasing time-activity curve, whereas gliomas graded as WHO grades 3 and 4 elicit an early activity peak around 10–20 min after injection, followed by a decrease of [^18^F]FET uptake (14, 15). Moreover, pre-treatment dynamic [^18^F]FET PET imaging has been proven to significantly increase the detection rate of more malignant foci (16, 17). However, the use of dynamic PET imaging using [^11^C]MET in the pre-treatment setting did not result in improved diagnostic accuracy in tumor grading (18). To explain these contradictory findings, Moulin-Romsée et al. discussed the role of the system-L-transporter (LAT) proteins, which are overexpressed on the membrane of glioma cells. Although the mechanism of FET accumulation in glioma cells remains unclear, LAT subtypes 1, 2, and 3 are believed to play a role. Glioma cells overexpress LAT-1, which also allows the transport of smaller amino acids such as methionine, and is primarily driven by intracellular amino acid concentrations and composition (19). Since FET is not metabolized intracellularly (20, 21), the inward transport of FET is believed to exceed the outward transport via LAT-1. Methionine, in contrast, is incorporated into proteins in the glioma cell, which is hypothesized to halt the inward transport, explaining the pre-treatment imaging results (18). To some extent, these mechanisms of action may also help us understand our findings of differences in the diagnostic accuracy of [^18^F]FET and [^11^C]MET PET imaging. Furthermore, it is known that differences in radiotracer distribution exist when comparing [^18^F]FET and [^11^C]MET, including greater uptake of [^11^C]MET in inflammatory lesions and different physiological uptake of [^11^C]MET in normal brain tissue compared to [^18^F]FET (20, 22, 23). Another striking finding concerned the non-significant difference in the TBR_mean_ values derived from the [^18^F]FET PET data when comparing patients with TP and those with TRA (*p* = 0.066). This is not in line with the available evidence reported in the most recent guidelines (9) and must be considered a consequence of the relatively limited sample size in this specific sub-cohort.

This study had several limitations, including the retrospective, single-center design, which inherently introduces a risk of selection bias and limits its generalizability. Second, the relatively small sample size reduces statistical power, leading to wider confidence intervals and, possibly, instability of the subgroup analyses. Due to the previously mentioned differences in radiotracer distribution and differences in the subsequent metabolic processes between [^18^F]FET and [^11^C]MET, no pooled analyses could be performed. Third, histopathology was not available in all patients and, although this is often the case in neuro-oncological research, this must be considered a limitation. Finally, dynamic PET protocols and reconstruction parameters may vary across centers, which could limit the generalizability of our findings. Despite these limitations, the results can provide clinically relevant guidance. The finding that static AA-PET parameters can be sufficient to differentiate TP from TRA implies that complex and time-consuming dynamic PET protocols may not be required in routine clinical practice. This can streamline imaging workflows, reduce patient burden, and facilitate timely therapeutic decision-making in neuro-oncology.

## Conclusion

This study suggests that dynamic AA-PET parameters do not provide significant added value in differentiating TP from TRA in patients with post-treatment glioma. While static AA-PET parameters demonstrated good-to-excellent diagnostic accuracy and may suffice for clinical decision-making, the current analysis did not formally assess the incremental value of using dynamic metrics in addition to static measures. The small cohort size, retrospective design, heterogeneous reference standard, and potential segmentation/measurement variability may have limited the power of the study to detect any modest but clinically relevant improvements when using dynamic imaging. Therefore, while dynamic imaging demonstrated no additional diagnostic value, this does not rule out the possibility of such an effect in a larger or more optimized study.

## Data Availability

The datasets generated and analyzed during the current study are available from the corresponding author on reasonable request. The data are not publicly available due to their containing information that could compromise the privacy of the participants. Requests to access the datasets should be directed to Dr. Dylan Henssen at dylan.henssen@medizin.uni-leipzig.de.

## References

[1] Louis DN Perry A Wesseling P Brat DJ Cree IA Figarella-Branger D The 2021 WHO classification of tumors of the central nervous system: a summary. Neuro Oncol. (2021) 23(8):1231–51. 10.1093/neuonc/noab106 34185076 PMC8328013

[2] Wen PY Macdonald DR Reardon DA Cloughesy TF Sorensen AG Galanis E Updated response assessment criteria for high-grade gliomas: response assessment in neuro-oncology working group. J Clin Oncol. (2010) 28(11):1963–72. 10.1200/JCO.2009.26.3541 20231676

[3] Brandsma D Stalpers L Taal W Sminia P van den Bent MJ. Clinical features, mechanisms, and management of pseudoprogression in malignant gliomas. Lancet Oncol. (2008) 9(5):453–61. 10.1016/S1470-2045(08)70125-6 18452856

[4] Young RJ Gupta A Shah AD Graber JJ Zhang Z Shi W Potential utility of conventional MRI signs in diagnosing pseudoprogression in glioblastoma. Neurology. (2011) 76(22):1918–24. 10.1212/WNL.0b013e31821d74e7 21624991 PMC3115805

[5] Albert NL Weller M Suchorska B Galldiks N Soffietti R Kim MM Response assessment in neuro-oncology working group and European Association for Neuro-Oncology recommendations for the clinical use of PET imaging in gliomas. Neuro Oncol. (2016) 18(9):1199–208. 10.1093/neuonc/now058 27106405 PMC4999003

[6] Santo G Laudicella R Linguanti F Nappi AG Abenavoli E Vergura V The utility of conventional amino acid PET radiotracers in the evaluation of glioma recurrence also in comparison with MRI. Diagnostics (Basel). (2022) 12(4). 10.3390/diagnostics12040844 35453892 PMC9027186

[7] Smits M. MRI biomarkers in neuro-oncology. Nat Rev Neurol. (2021) 17(8):486–500. 10.1038/s41582-021-00510-y 34149051

[8] Galldiks N Kaufmann TJ Vollmuth P Lohmann P Smits M Veronesi MC Challenges, limitations and pitfalls of PET and advanced MRI in patients with brain tumors – a report of the PET/RANO group. Neuro Oncol. (2024) 26(7):1181–94. 10.1093/neuonc/noae049 38466087 PMC11226881

[9] Law I Albert NL Arbizu J Boellaard R Drzezga A Galldiks N Joint EANM/EANO/RANO practice guidelines/SNMMI procedure standards for imaging of gliomas using PET with radiolabelled amino acids and [(18)F]FDG: version 1.0. Eur J Nucl Med Mol Imaging. (2019) 46(3):540–57. 10.1007/s00259-018-4207-9 30519867 PMC6351513

[10] Leao DJ Craig PG Godoy LF Leite CC Policeni B. Response assessment in neuro-oncology criteria for gliomas: practical approach using conventional and advanced techniques. AJNR Am J Neuroradiol. (2020) 41(1):10–20. 10.3174/ajnr.A6358 31857322 PMC6975322

[11] Mandrekar JN. Receiver operating characteristic curve in diagnostic test assessment. J Thorac Oncol. (2010) 5(9):1315–6. 10.1097/JTO.0b013e3181ec173d 20736804

[12] Simundic AM. Measures of diagnostic accuracy: basic definitions. EJIFCC. (2009) 19(4):203–11. 27683318 PMC4975285

[13] Kebir S Fimmers R Galldiks N Schafer N Mack F Schaub C Late pseudoprogression in glioblastoma: diagnostic value of dynamic O-(2-[18F]fluoroethyl)-L-tyrosine PET. Clin Cancer Res. (2016) 22(9):2190–6. 10.1158/1078-0432.CCR-15-1334 26673798

[14] Lohmann P Herzog H Rota Kops E Stoffels G Judov N Filss C Dual-time-point O-(2-[(18)F]fluoroethyl)-L-tyrosine PET for grading of cerebral gliomas. Eur Radiol. (2015) 25(10):3017–24. 10.1007/s00330-015-3691-6 25813014

[15] Popperl G Kreth FW Mehrkens JH Herms J Seelos K Koch W FET PET for the evaluation of untreated gliomas: correlation of FET uptake and uptake kinetics with tumour grading. Eur J Nucl Med Mol Imaging. (2007) 34(12):1933–42. 10.1007/s00259-007-0534-y 17763848

[16] Jansen NL Graute V Armbruster L Suchorska B Lutz J Eigenbrod S MRI-suspected low-grade glioma: is there a need to perform dynamic FET PET? Eur J Nucl Med Mol Imaging. (2012) 39(6):1021–9. 10.1007/s00259-012-2109-9 22491781

[17] Kunz M Thon N Eigenbrod S Hartmann C Egensperger R Herms J Hot spots in dynamic (18)FET-PET delineate malignant tumor parts within suspected WHO grade II gliomas. Neuro Oncol. (2011) 13(3):307–16. 10.1093/neuonc/noq196 21292686 PMC3064604

[18] Moulin-Romsée G D'Hondt E de Groot T Goffin J Sciot R Mortelmans L Non-invasive grading of brain tumours using dynamic amino acid PET imaging: does it work for 11C-methionine? Eur J Nucl Med Mol Imaging. (2007) 34(12):2082–7. 10.1007/s00259-007-0557-4 17763978

[19] van der Kolk AG Henssen D Schroeder HW III Hall LT. In PET Agents for Primary Brain Tumor Imaging. Brisbane: Exon Publications (2023). 10.36255/pet-agents-for-primary-brain-tumor-imaging 38471020

[20] Ishiwata K Kubota K Murakami M Kubota R Sasaki T Ishii S Re-evaluation of amino acid PET studies: can the protein synthesis rates in brain and tumor tissues be measured *in vivo*? J Nucl Med. (1993) 34(11):1936–43. 8229238

[21] Wester HJ Herz M Weber W Heiss P Senekowitsch-Schmidtke R Schwaiger M Synthesis and radiopharmacology of O-(2-[18F]fluoroethyl)-L-tyrosine for tumor imaging. J Nucl Med. (1999) 40(1):205–12. 9935078

[22] Hashimoto S Inaji M Nariai T Kobayashi D Sanjo N Yokota T Usefulness of [(11)C] methionine PET in the differentiation of tumefactive multiple sclerosis from high grade astrocytoma. Neurol Med Chir (Tokyo). (2019) 59(5):176–83. 10.2176/nmc.oa.2018-0287 30996153 PMC6527963

[23] Stober B Tanase U Herz M Seidl C Schwaiger M Senekowitsch-Schmidtke R. Differentiation of tumour and inflammation: characterisation of [methyl-3H]methionine (MET) and O-(2-[18F]fluoroethyl)-L-tyrosine (FET) uptake in human tumour and inflammatory cells. Eur J Nucl Med Mol Imaging. (2006) 33(8):932–9. 10.1007/s00259-005-0047-5 16604346

